# Supplementary material for: A cross-sectional study on the impact of the COVID-19 pandemic on psychological outcomes: Multiple indicators and multiple causes modeling
Source: PLoS One. 2022 Nov 9;17(11):e0277368. doi: 10.1371/journal.pone.0277368 (PMC9645638; doi:10.1371/journal.pone.0277368)
Supplement: S1 Table — (DOC) [file pone.0277368.s002.doc]

**S1 Table. Descriptive and Confirmatory Statistics for Impact of Event Scale-Revised**

|  | **Descriptive Statistics** | | | | | | **Confirmatory Factor Analysis** | | | | | | |  |
| --- | --- | --- | --- | --- | --- | --- | --- | --- | --- | --- | --- | --- | --- | --- |
|  | **M** | **SD** | **Skew** | | **Kurt** | **α** | **B** | **S.E** | **C.R** | **P** | **λ** | **SMC** | **Com** | **AVE** |
| **Intrusion** | | | | |  |  |  |  |  |  |  |  |  |  |
| **Q1_Int** | 1.09 | 1.04 | .872 | | 0.208 | 0.871 | 1.000 |  |  |  | 0.655 | 0.429 | .0884 | 0. 604 |
| **Q2_Int** | 0.87 | 1.11 | 1.24 | | 0.750 | 0.876 | x | x | x | x | x | x |  |  |
| **Q3_Int** | 1.23 | 1.09 | 0.746 | | -.159 | 0.860 | 1.268 | 0.051 | 25.074 | *** | 0.776 | 0.603 |  |  |
| **Q6_Int** | 0.98 | 1.07 | 0.991 | | 0.304 | 0.861 | 1.285 | 0.059 | 21.813 | *** | 0.802 | 0.644 |  |  |
| **Q9_Int** | 0.86 | 1.07 | 1.19 | | 0.664 | 0.863 | 1.280 | 0.059 | 21.668 | *** | 0.796 | 0.633 |  |  |
| **Q14_Int** | 0.86 | 1.04 | 1.13 | | 0.608 | 0.870 | x | x | x | x | x | x |  |  |
| **Q16_Int** | 0.91 | 1.05 | 1.09 | | 0.551 | 0.856 | 1.349 | 0.059 | 22.711 | *** | 0.845 | 0.714 |  |  |
| **Q20_Int** | 0.41 | 0.890 | 2.36 | | 5.056 | 0.878 | x | x | x | x | x | x |  |  |
|  | **0.90** | **1.045** |  | |  | 0.882 |  |  |  |  |  |  |  |  |
| **Avoidance** | | | | |  |  |  |  |  |  |  |  |  |  |
| **Q5_Avo** | 1.29 | 1.24 | 0.707 | | -0.521 | .854 | 1.177 | 0.075 | 15.694 | *** | 0.621 | 0.385 | 0.870 | 0.500 |
| **Q7_Avo** | 0.93 | 1.14 | 1.16 | | 0.492 | 0.858 | 1.00 | - | - | *** | 0.576 | 0.331 |  |  |
| **Q8_Avo** | 0.99 | 1.17 | 1.05 | | 0.175 | 0.845 | 1.239 | 0.074 | 16.825 | *** | 0.687 | 0.472 |  |  |
| **Q11_Avo** | 1.20 | 1.18 | 0.780 | | -0.304 | 0.833 | 1.447 | 0.079 | 18.302 | *** | 0.785 | 0.617 |  |  |
| **Q12_Avo** | 0.99 | 1.07 | 0.973 | | 0.286 | 0.845 | x | x | x | x | x | x |  |  |
| **Q13_Avo** | 1.03 | 1.07 | 0.895 | | 0.128 | 0.851 | 1.016 | 0.065 | 15.711 | *** | 0.621 | 0.386 |  |  |
| **Q17_Avo** | 0.98 | 1.17 | 1.07 | | 0.217 | 0.833 | 1.487 | 0.080 | 18.681 | *** | 0.814 | 0.662 |  |  |
| **Q22_Avo** | 0.94 | 1.12 | 1.10 | | 0.414 | 0.839 | 1.337 | 0.074 | 18.044 | *** | 0.767 | 0.588 |  |  |
| **Q5_Avo** | 1.29 | 1.24 | .707 | | -0.521 | 0.854 | x | x | x | x | x | x |  |  |
|  | **1.07** | **1.15** |  | |  | **0.862** |  |  |  |  |  |  |  |  |
| **Hyperarousal** | | | | |  |  |  |  |  |  |  |  |  |  |
| **Q4_Hyp** | 0.97 | 1.08 | 1.01 | | .268 | 0.793 | 0.819 | 0.029 | 27.820 | *** | 0.715 | 0.512 | 0.816 | 0.528 |
| **Q10_Hyp** | 0.79 | 1.03 | 1.23 | | .753 | 0.806 | 1.00 | - | - | *** | 0.818 | 0.668 |  |  |
| **Q15_Hyp** | 1.04 | 1.24 | 1.05 | | .029 | 0.806 | x | x | x | x | x | x |  |  |
| **Q18_Hyp** | 1.11 | 1.21 | 0.913 | | -.147 | 0.783 | x | x | x | x | x | x |  |  |
| **Q19_Hyp** | 0.55 | 0.961 | 1.83 | | 2.701 | 0.808 | 0.752 | 0.026 | 29.181 | *** | 0.736 | 0.542 |  |  |
| **Q21_Hyp** | 1.04 | 1.22 | 1.02 | | .064 | 0.831 | 0.797 | 0.035 | 22.597 | *** | 0.623 | 0.388 |  |  |
|  | **0.91** | **1.12** |  | |  | **0.832** |  |  |  |  |  |  |  |  |
|  | **Relationship** | | | | | |  |  |  |  |  |  |  |  |
|  | **Intrusion** | | | **Avoidance** | | | 0.375 | 0.028 | 13.512 | *** | 0.768 |  |  |  |
|  | **Hyperarousal** | | | **Avoidance** | | | 0.588 | 0.033 | 18.072 | *** | 0.865 |  |  |  |
|  | **Intrusion** | | | **Hyperarousal** | | | 0.701 | 0.030 | 23.056 | *** | 0.977 |  |  |  |
|  | **e1** | | | **e3** | | | 0.179 | 0.023 | 7.782 | *** | 0.292 |  |  |  |

M = Mean, SD = Standard Deviation, Skew = Skewness, Kurt = Kurtosis, α = Alpha, B = un-standardized estimates, S.E = Stander Error, C.R = Critical Ratio, P = probability, λ = load-ing, SMC = Squared Multiple Correlation, Com = Composite Reliability, AVE = Average Variance Extracted, x = items removed from confirmatory factor analysis (CFA), *** significance at .001.
