# Supplementary material for: A cross-sectional study on the impact of the COVID-19 pandemic on psychological outcomes: Multiple indicators and multiple causes modeling
Source: PLoS One. 2022 Nov 9;17(11):e0277368. doi: 10.1371/journal.pone.0277368 (PMC9645638; doi:10.1371/journal.pone.0277368)
Supplement: S2 Table — (DOC) [file pone.0277368.s003.doc]

**S2 Table. Descriptive and Confirmatory Statistics for Psychological Outcomes**

|  | **Descriptive Statistics** | | | | | **Confirmatory Factor Analysis** | | | | | | |  |
| --- | --- | --- | --- | --- | --- | --- | --- | --- | --- | --- | --- | --- | --- |
|  | **M** | **SD** | **Skew** | **Kurt** | **α** | **un** | **SE** | **C.R** | **p** | **λ** | **SMC** | **AVE** | **Com** |
| **Depression** | | | |  |  |  |  |  |  |  |  |  |  |
| Dep1 | 1.24 | 1.00 | 0.439 | -0.857 | 0.873 | 1.00 | - | - | - | 0.627 | 0.393 | **0.481** | 0.882 |
| Dep2 | 0.89 | 0.921 | 0.858 | -.093 | 0.861 | 1.164 | 0.057 | 20.456 | *** | 0.795 | 0.633 |  |  |
| Dep3 | 1.21 | 1.08 | 0.432 | -1.10 | 0.872 | 1.199 | 0.065 | 18.463 | *** | 0.693 | 0.481 |  |  |
| Dep4 | 1.08 | 0.974 | 0.631 | -0.563 | 0.864 | 1.148 | 0.059 | 19.422 | *** | 0.741 | 0.549 |  |  |
| Dep5 | 0.91 | 1.00 | 0.817 | -0.499 | 0.870 | 1.067 | 0.060 | 17.908 | *** | 0.667 | 0.445 |  |  |
| Dep6 | 0.80 | 0.991 | 1.06 | -0.020 | 0.864 | 1.137 | 0.060 | 19.052 | *** | 0.722 | 0.522 |  |  |
| Dep7 | 0.88 | 0.988 | 0.868 | -0.350 | 0.865 | 1.109 | 0.059 | 18.734 | *** | 0.707 | 0.499 |  |  |
| Dep8 | 0.49 | 0.822 | 1.69 | 2.04 | 0.874 | 0.779 | 0.048 | 16.358 | *** | 0.596 | 0.356 |  |  |
| Dep9 | 0.32 | 0.711 | 2.42 | 5.30 | 0.880 | r | x | x | x | x | x |  |  |
| Mean | **0.86** | **0.94** |  |  | **0.882** |  |  |  |  |  |  |  |  |
| **Anxiety** | | | |  |  |  |  |  |  |  |  |  |  |
| Anx1 | 1.10 | 0.992 | 0.647 | -0.581 | 0.861 | 1.00 | - | - | - | 0.705 | 0.497 | 0.**546** | 0.893 |
| Anx2 | 0.72 | 0.888 | 1.14 | 0.503 | 0.857 | 0.973 | 0.043 | 22.669 | *** | 0.766 | 0.587 |  |  |
| Anx3 | 1.07 | 0.944 | 0.684 | -0.355 | 0.856 | 1.071 | 0.046 | 23.434 | *** | 0.794 | 0.630 |  |  |
| Anx4 | 0.89 | 0.952 | 0.889 | -0.144 | 0.858 | 1.034 | 0.046 | 22.464 | *** | 0.759 | 0.576 |  |  |
| Anx5 | 0.64 | 0.842 | 1.19 | 0.609 | 0.861 | 0.895 | 0.041 | 22.001 | *** | 0.743 | 0.552 |  |  |
| Anx6 | 0.88 | 0.939 | 0.857 | -0.193 | 0.863 | 0.982 | 0.045 | 21.670 | *** | 0.731 | 0.535 |  |  |
| Anx7 | 1.05 | 0.968 | 0.726 | -0.385 | 0.866 | 0.920 | 0.047 | 19.763 | *** | 0.665 | 0.442 |  |  |
| Mean | **0.907** | **0.932** |  |  | **0.860** |  |  |  |  |  |  |  |  |
| **Insomnia** | | | |  |  |  |  |  |  |  |  |  |  |
| Ins1 | 1.13 | 1.15 | 0.799 | -0.267 | 0.880 | 1.00 | - | - | - | 0.749 | 0.560 | **0.572** | 0.902 |
| Ins2 | 0.89 | 1.057 | 1.08 | 0.423 | 0.889 | 0.817 | 0.030 | 27.540 | *** | 0.667 | 0.444 |  |  |
| Ins3 | 1.21 | 1.297 | 0.807 | -0.507 | 0.903 | 0.893 | 0.048 | 18.623 | *** | 0.594 | 0.353 |  |  |
| Ins4 | 1.85 | 1.198 | 0.142 | -0.905 | 0.879 | 1.094 | 0.043 | 25.339 | *** | 0.788 | 0.620 |  |  |
| Ins5 | 1.49 | 0.834 | 1.614 | 1.59 | 0.892 | 0.700 | 0.030 | 23.091 | *** | 0.724 | 0.524 |  |  |
| Ins6 | 1.09 | 1.18 | 0.923 | -0.100 | 0.873 | 1.198 | 0.042 | 28.334 | *** | 0.871 | 0.759 |  |  |
| Ins7 | 1.27 | 1.22 | 0.679 | -0.585 | 0.876 | 1.222 | 0.044 | 27.972 | *** | 0.861 | 0.741 |  |  |
| Mean | **1.27** | **1.13** |  |  | **0.900** |  |  |  |  |  |  |  |  |
|  | **Relationship** | | | | |  |  |  |  |  |  |  |  |
|  | **Anxiety** | | **Depression** | | | 0.345 | 0.026 | 13.321 | *** | 0.786 |  |  |  |
|  | **Anxiety** | | **Insomnia** | | | 0.369 | 0.029 | 12.862 | *** | 0.612 |  |  |  |
|  | **Depression** | | **Insomnia** | | | 0.410 | 0.030 | 13.469 | *** | 0.756 |  |  |  |
|  | e16 | | e17 | | | 0.253 | 0.023 | 10.923 | *** | 0.421 |  |  |  |

M = Mean, SD = Standard Deviation, Skew = Skewness, Kurt = Kurtosis, α = Alpha, B = un-standardized estimates, S.E = Stander Error, C.R = Critical Ratio, P = probability, λ = load-ing, SMC = Squared Multiple Correlation, Com = Composite Reliability, AVE = Average Variance Extracted, x = items removed from confirmatory factor analysis (CFA), *** significance at .001.
