# Supplementary material for: A cross-sectional study on the impact of the COVID-19 pandemic on psychological outcomes: Multiple indicators and multiple causes modeling
Source: PLoS One. 2022 Nov 9;17(11):e0277368. doi: 10.1371/journal.pone.0277368 (PMC9645638; doi:10.1371/journal.pone.0277368)
Supplement: S3 Table — (DOC) [file pone.0277368.s004.doc]

| **S3 Table. Results of Differential Item Functioning (DIF)** | | | | | | | |
| --- | --- | --- | --- | --- | --- | --- | --- |
| **Group** | | **B** | **S.E** | **C.R** | **p** | **Decision** | **λ** |
| **Depression** |  |  |  |  |  |  |  |
| Dep2 | NO PTSD | -0.060 | 0.080 | -0.755 | 0.450 | Matching | 0.031 |
|  | PTSD | 0.060 | 0.080 | 0.755 | 0.450 |  | -0.031 |
| Dep 3 | NO PTSD | -0.043 | 0.092 | -0.473 | 0.636 | Matching | 0.019 |
|  | PTSD | 0.043 | 0.092 | 0.473 | 0.636 |  | -0.019 |
| Dep 4 | NO PTSD | -0.015 | 0.084 | -0.173 | 0.863 | Matching | 0.007 |
|  | PTSD | 0.015 | 0.084 | 0.173 | 0.863 |  | -0.007 |
| Dep 5 | NO PTSD | -0.102 | 0.083 | -1.218 | 0.223 | Matching | 0.048 |
|  | PTSD | 0.102 | 0.083 | 1.218 | 0.223 |  | -0.048 |
| Dep 6 | NO PTSD | -0.115 | 0.083 | -1.381 | 0.167 | Matching | 0.056 |
|  | PTSD | 0.115 | 0.083 | 1.381 | 0.167 |  | -0.056 |
| Dep 7 | NO PTSD | -0.175 | 0.081 | -2.158 | 0.031 | DIF | 0.085 |
|  | PTSD | 0.175 | 0.081 | 2.158 | 0.031 |  | -0.085 |
| Dep 8 | NO PTSD | -0.184 | 0.065 | -2.832 | 0.005 | DIF | 0.108 |
|  | PTSD | 0.184 | 0.065 | 2.832 | 0.005 |  | -0.108 |
| **Anxiety** | |  |  |  |  |  |  |
| Anx2 | NO PTSD | -0.023 | 0.069 | -0.331 | 0.741 | Matching | 0.012 |
|  | PTSD | 0.023 | 0.069 | 0.331 | 0.741 |  | -0.012 |
| Anx 3 | NO PTSD | -0.021 | 0.074 | -0.283 | 0.777 | Matching | -0.011 |
|  | PTSD | 0.021 | 0.074 | 0.283 | 0.777 |  | 0.011 |
| Anx 4 | NO PTSD | -0.022 | 0.074 | -0.293 | 0.770 | Matching | -0.071 |
|  | PTSD | 0.022 | 0.074 | 0.293 | 0.770 |  | -0.011 |
| Anx 5 | NO PTSD | -0.083 | 0.064 | -1.298 | 0.194 | Matching | 0.047 |
|  | PTSD | 0.083 | 0.064 | 1.298 | 0.194 |  | -0.047 |
| Anx 6 | NO PTSD | -0.086 | 0.072 | -1.198 | 0.231 | Matching | 0.044 |
|  | PTSD | 0.086 | 0.072 | 1.198 | 0.231 |  | -0.044 |
| Anx 7 | PTSD | -0.110 | 0.073 | -1.502 | 0.133 | Matching | 0.055 |
|  | NO PTSD | 0.110 | 0.073 | 1.502 | 0.133 |  | -0.055 |
| **Insomnia** | |  |  |  |  |  |  |
| Ins 2 | NO PTSD | -0.114 | 0.061 | -1.887 | 0.059 | Matching | 0.052 |
|  | PTSD | 0.114 | 0.061 | 1.887 | 0.059 |  | -0.052 |
| Ins 3 | NO PTSD | -0.129 | 0.098 | -1.321 | 0.187 | Matching | 0.048 |
|  | PTSD | 0.129 | 0.098 | 1.321 | 0.187 |  | -0.048 |
| Ins 4 | NO PTSD | -0.178 | 0.091 | -1.944 | 0.052 | Matching | -0.071 |
|  | PTSD | 0.178 | 0.091 | 1.944 | 0.052 |  | 0.071 |
| Ins 5 | NO PTSD | -0.174 | 0.065 | -2.700 | 0.007 | DIF | -0.100 |
|  | PTSD | 0.174 | 0.065 | 2.700 | 0.007 |  | 0.100 |
| Ins 6 | NO PTSD | -0.177 | 0.089 | -1.976 | 0.048 | DIF | -0.072 |
|  | PTSD | 0.177 | 0.089 | 1.976 | 0.048 |  | 0.072 |
| Ins 7 | NO PTSD | -0.005 | 0.090 | -0.059 | 0.953 | Matching | 0.002 |
|  | PTSD | 0.005 | 0.090 | 0.059 | 0.953 |  | -0.002 |

PTSD = Post traumatic stress disorder, B = unstandardized estimates, S.E = Stander Error, C.R = Critical Ratio, P = probability = loading
