# Supplementary material for: A cross-sectional study on the impact of the COVID-19 pandemic on psychological outcomes: Multiple indicators and multiple causes modeling
Source: PLoS One. 2022 Nov 9;17(11):e0277368. doi: 10.1371/journal.pone.0277368 (PMC9645638; doi:10.1371/journal.pone.0277368)
Supplement: S4 Table — (DOC) [file pone.0277368.s005.doc]

**S4 Table****. Items of Impact of Event Scale-Revised for Covid-19**

| Symbols | Items \Statement\ Questions |
| --- | --- |
| Q1_Int | Any reminder brought back feelings about it. |
| Q2_Int | I had trouble staying asleep. |
| Q3_Int | Other things kept making me think about it. |
| Q4_Hyp | I felt irritable and angry. |
| Q5_Avo | I avoided letting myself get upset when I thought about it or was reminded of it. |
| Q6_Int | I thought about it when I didn’t mean to. |
| Q7_Avo | I felt as if it hadn’t happened or wasn’t real. |
| Q8_Avo | I stayed away from reminders of it. |
| Q9_Int | Pictures about it popped into my mind. |
| Q10_Hyp | I was jumpy and easily startled. |
| Q11_Avo | I tried not to think about it. |
| Q12_Avo | I was aware that I still had a lot of feelings about it, but I didn’t deal with them. |
| Q13_Avo | My feelings about it were kind of numb. |
| Q14_Int | I found myself acting or feeling like I was back at that time. |
| Q15_Hyp | I had trouble falling asleep. |
| Q16_Int | I had waves of strong feelings about it. |
| Q17_Avo | I tried to remove it from my memory. |
| Q18_Hyp | I had trouble concentrating. |
| Q19_Hyp | Reminders of it caused me to have physical reactions, such as sweating, trouble breathing, nausea, or a pounding heart. |
| Q20_Int | I had dreams about it. |
| Q21_Hyp | I felt watchful and on-guard. |
| Q22_Avo | I tried not to talk about it. |
| Int =Intrusion, Avo =Avoidance, Hyp =Hyperarousal subscale | |
