# Supplementary material for: A cross-sectional study on the impact of the COVID-19 pandemic on psychological outcomes: Multiple indicators and multiple causes modeling
Source: PLoS One. 2022 Nov 9;17(11):e0277368. doi: 10.1371/journal.pone.0277368 (PMC9645638; doi:10.1371/journal.pone.0277368)
Supplement: Attachment — Submitted filename: Response to Reviewers.docx [file pone.0277368.s006.docx]

**Dear Prof Dr. Muhammad Shahzad Aslam,**

**Academic Editor,**

**PLOS ONE,**

We want to provide our extreme thanks and appreciation to you and the reviewers for your time and immense efforts in handling and reviewing our manuscript.

Dear Prof, we are delighted to submit the revised manuscript entitled “**Impact of COVID-19 pandemic on the psychological outcomes: multiple indicators and multiple causes (MIMIC) modeling**” to be considered for publication in your Journal within the category of an original research paper. We are grateful to you and the reviewers for your insightful comments on our paper. We have implemented all the Journal Requirements and reviewers’ comments and recommendations with extreme happiness. Updates are highlighted in yellow in the current revised manuscript. Also, here is our Point-by-point response to reviewers; we provided point-by-point responses to the editor and reviewers' comments and concerns. Further, please find a revised manuscript for our paper submitted to the journal via the online submission system.

**Journal Requirements:**

When submitting your revision, we need you to address these additional requirements.

1. Please ensure that your manuscript meets PLOS ONE's style requirements, including those for file naming. The PLOS ONE style templates can be found at https://journals.plos.org/plosone/s/file?id=wjVg/PLOSOne_formatting_sample_main_body.pdf and <https://journals.plos.org/plosone/s/file?id=ba62/PLOSOne_formatting_sample_title_authors_affiliations.pdf>.

**Response:**

Thank you very much for your time and consideration in handling and checking our manuscript, and we greatly appreciate it. We have checked PLOS ONE's style requirements, including those for file naming, PLOS ONE style templates and we follow it in the current revised manuscript.

2. Please amend your current ethics statement to address the following concerns:

a) Did participants provide their written or verbal informed consent to participate in this study?

b) If consent was verbal, please explain i) why written consent was not obtained, ii) how you documented participant consent, and iii) whether the ethics committees/IRB approved this consent procedure.

**Response:**

Thank you very much for your comment, we have added the Ethics and consent statement section to the methodology on page 5; lines 96-101, and as stated we obtained an electronic informed consent via a Google form from all participants who agree to participate in this study. We obtained an electronic informed consent via a Google form because the contact was not feasible due to the lockdown, movement control order, the spread of COVID 19, and as a part of the infection control during the data collection of this study which was online also due to the same reasons.

3. We note that you have indicated that data from this study are available upon request. PLOS only allows data to be available upon request if there are legal or ethical restrictions on sharing data publicly. For information on unacceptable data access restrictions, please see http://journals.plos.org/plosone/s/data-availability#locunacceptable-data-access-restrictions. In your revised cover letter, please address the following prompts:

a) If there are ethical or legal restrictions on sharing a de-identified data set, please explain them in detail (e.g., data contain potentially identifying or sensitive patient information) and who has imposed them (e.g., an ethics committee). Please also provide contact information for a data access committee, ethics committee, or other institutional body to which data requests may be sent.

**Response:**

Thank you very much for your note, yes we indicated that data from this study are available upon request for academic and research purposes because we have stated in the consent form for the participants that their response is confidential and will protect throughout the data collection and analysis to guarantee data integrity and privacy. We have stated also the data is for exclusive scientific use and will not be revealed to any third party only for research and academic purpose upon any request for that. We have provided the contact information for the data access request for any research and academic purpose in our cover letter for submitting our revised manuscript.

4. Your ethics statement should only appear in the Methods section of your manuscript. If your ethics statement is written in any section besides the Methods, please move it to the Methods section and delete it from any other section. Please ensure that your ethics statement is included in your manuscript, as the ethics statement entered into the online submission form will not be published alongside your manuscript.

**Response:**

Thank you very much for your comment, we have moved, and included the ethics statement in the Methods section and deleted it from the sections of the declaration.

| **Reviewer 1**  Many thanks for giving me the opportunity to review. It is an interesting article where authors studied the psychological impact of COVID-19 pandemic in a multi-country setting. There are the following points that need to be addressed before acceptance of the manuscript.  **Response:**  Thank you very much **Dr. Bushra Khan** for your time and great efforts in reviewing our manuscript and for your positive feedback, valuable suggestions, comments, and recommendations to improve the quality of our paper. We have addressed all the concerns raised in the current revised manuscript and this letter; our point-by-point responses will be presented. | | |
| --- | --- | --- |
| **Sections** | **Comments** | **Responses** |
| **Methods** | | |
| 1. | Please include the exact duration and time when the study was conducted. | Thank you very much for raising this point, we have added the exact date for the data collection from 01/04/2020 to 16/05/2020 in the Participants and Procedure section under the Methodology Page 5; line 105. |
| 2. | It seems that the study participants had diverse socio-demographic characteristics, please also include the age  group to add more clarity. | We appreciate your efforts in raising this important point for more clarity, we have added the results of participants' age group to the Demographic characteristics results on Page 8&9; lines 193-198, and to table 1 for Socio-demographic characteristics of participants on page 9. The results revealed that the mean age of the participants was 33.06, Std. D= 9.3; distributed according to the following age categories 403(40.3%) their age was between 24-35 years and 141(35.0%) of them experienced PTSD, 254 (25.4%) aged 36-45 years, and 91(35.8%) of them diagnosed with PTSD, following by 242 (24.2%) of the participants aged 18-25 and 101(41.7%) of them experienced PTSD, and 100 (10%) their age was between 46-75 years old and 27(27.0%) of them diagnosed with PTSD. |
| 3. | It is mentioned that participants were recruited via social media, please explain what types of social media sources were used. The Procedure section is missing. I believe such information can be included in it. | Thank you for raising this point to make the section more informative, We have added the type of social media sources we used; WhatsApp, Facebook, and emails to recruit the participants as the face-to-face contact and physical distribution of the questionnaire were not feasible due to the lockdown, movement control order, the spread of COVID 19, and as a part of the infection control during the data collection time for the current study. We have also added the Procedure section under the methodology named Participants and Procedure including more information under it regarding the participants and procedure as you suggested, Page 5; lines 102-112. |
| 4&5 | This study was conducted in a multi-country setting with people of diverse socio-demographic characteristics. It would be helpful if the authors could explain if they translated/adapted the instruments in different languages and how did they conduct that process. Moreover, using psychological instruments cross-culturally is bit challenging, it would be helpful if they could mention the way they managed response bias due to the language barrier. | Thank you very much for your comment, we have distributed the questionnaire in English regardless of participants’ country background. Thus, we did not translate or adapt the survey question in different languages. We conducted a pilot study before the actual data collection to make sure of the clarity and readability of the questionnaire items. The pilot study helped us ensure that the item descriptions could be understood by the target participants. Moreover, we provided clear instructions on the introduction page of the Google Form survey and in each section of the questionnaire regarding the answer/fill-in of the survey and we provide them with researchers’ contact details for any clarification or help if they face any difficulties. However, we acknowledged that the participants might have different levels of English and thus we have added it as one of the limitations of our study and as a recommendation for future research on page 17, lines; 382-389. |
| **Reviewer 2:** | | |
| **Sections** | **Comments** | **Responses** |
| **Introduction** | Introduction study and the relevance of the findings. The introduction is a little short and would benefit from some rephrasing and restructuring. In addition, the hypotheses should be stated in the introduction instead of in the results. | Thank you very much **Dr. Alishba Hania** for your efforts and positive feedback. we appreciate how thoroughly you reviewed and checked the manuscript sections. |
| **Method** | Full details on how the study was actually carried out is mentioned. | Thank you for your efforts and positive feedback. |
| **Results** | Clearly reveals what actually occurred to the subjects. The results explain the statistical analysis shown in related tables, diagrams and graphs. | Thank you very much for your positive feedback. |
| **Discussion** | It includes an absolute comparison of what is already identified in the topic of interest and the clinical relevance of what has been newly established. A discussion on a possible related limitations and necessitation for further studies should also be indicated. Conclusions should ensure that recommendations stated are suitable for the results attained within the capacity of the study. The authors should also concentrate on the limitations in the study and their effects on the outcomes and the proposed suggestions for future studies | Thank you for the guidance. We have now elaborated on how future studies can be conducted with the consideration of the present study’s limitations, pages 18&19; lines 382-398. Regarding the Conclusions section, we have toned down our statement to fit the capacity of the present study, page 19; lines 410-419. |

**We thank you again for your immense efforts and positive feedback.**

**Sincerely yours,**
