# Supplementary material for: A cross-sectional study on the impact of the COVID-19 pandemic on psychological outcomes: Multiple indicators and multiple causes modeling
Source: PLoS One. 2022 Nov 9;17(11):e0277368. doi: 10.1371/journal.pone.0277368 (PMC9645638; doi:10.1371/journal.pone.0277368)
Supplement: Attachment — Submitted filename: Response to Academic Editor.docx [file pone.0277368.s007.docx]

**Dear Prof Dr. Muhammad Shahzad Aslam,**

**Academic Editor,**

**PLOS ONE,**

First, we express our extreme gratitude for your extraordinary efforts and incredible handling and reviewing of our revised manuscript after implementing the valuable and constructive Editor's and reviewers' comments and suggestions raised in the first round of the revision to improve the accuracy and level of the manuscript; we greatly appreciate it.

Dear Prof, we have received your decision on the revised manuscript with extreme happiness and respect. Regarding your additional minor comment on the revised manuscript version, we are delighted to submit the revised manuscript R2 entitled “**A cross-sectional study on the impact of the COVID-19 pandemic on psychological outcomes: multiple indicators and multiple causes modeling**” after implementing your comment to provide strobe statement as per cross-sectional study and to prepare the manuscript according to strobe guideline and submit the STROBE checklist as a supplementary file. Accordingly, we have prepared our revised manuscript according to the strobe statement and submitted the checklist as a supplementary file. Moreover, We have checked and reviewed the reference list and ensured that it is complete and correct. In addition, we have done proofreading for the entire manuscript.

We are grateful to you, and We have implemented the Journal Requirement and Editor's comment with extreme happiness. Updates are highlighted in yellow in the second revised manuscript. Also, here is our Response to the academic editor.

***Thank you again for your extraordinary efforts and wonderful and constructive editor of our manuscript.***

**We greatly appreciate it.**

**Journal Requirements:**

Please review your reference list to ensure that it is complete and correct

**Response:**

Thank you very much for your time and consideration in handling and checking our manuscript; we greatly appreciate it. We have checked and reviewed the reference list and ensured that it is complete and correct.

**Additional Editor Comments:**

Please Provide strobe statement as per cross sectional study and prepare your manuscript according to strobe guideline and submit the checklist as per STROBE criteria and attached as supplementary file.

**Response:**

Thank you very much for your comment. We have prepared our revised manuscript according to the strobe statement and submitted the checklist as a supplementary file

**Thank you very much once again for your extraordinary efforts and incredible handling and reviewing of our manuscript,**

**We highly appreciate it.**

**Yours sincerely,**
